# Validation of the French Version of the Child Post-Traumatic Stress Reaction Index: Psychometric Properties in French Speaking School-Aged Children

**DOI:** 10.1371/journal.pone.0112603

**Published:** 2014-12-02

**Authors:** Bertrand Olliac, Philippe Birmes, Eric Bui, Charlotte Allenou, Alain Brunet, Isabelle Claudet, Jérôme Sales de Gauzy, Hélène Grandjean, Jean-Philippe Raynaud

**Affiliations:** 1 Institut National de la Santé et de la Recherche Médicale Unité Mixte de Recherche 1094, Tropical Neuroepidemiology, Limoges, France; 2 Univ Limoges, School of Medicine, Institute of Neuroepidemiology and Tropical Neurology, Centre National de la Recherche Scientifique FR 3503 GEIST, Limoges, France; 3 Institut National de la Santé et de la Recherche Médicale Unité Mixte de Recherche 1027, Université Paul Sabatier, Toulouse, France; 4 Pôle Hospitalo-Universitaire de Psychiatrie de l'enfant et de l'adolescent, Centre Hospitalier Esquirol, Limoges, France; 5 Université de Toulouse, Université Paul Sabatier, Laboratoire du Stress Traumatique (LST – EA 4560) Centre Hospitalier Universitaire de Toulouse, Hôpital Casselardit, Toulouse, France; 6 Massachusetts General Hospital & Harvard Medical School, Boston, Massachusetts, United States of America; 7 McGill University and Douglas Mental Health University Institute, Montréal, Quebec, Canada; 8 Unité des Urgences Pédiatriques, Hôpital des Enfants, Centre Hospitalier Universitaire de Toulouse, Toulouse, France; 9 Service Chirurgie Orthopédique, Traumatologique et Plastique, Hôpital des Enfants, Centre Hospitalier Universitaire de Toulouse, Toulouse, France; 10 Institut National de la Santé et de la Recherche Médicale Unité Mixte de Recherche 1027, Université Paul Sabatier, Toulouse, France; 11 Institut National de la Santé et de la Recherche Médicale Unité Mixte de Recherche 1027, Université Paul Sabatier, CHU de Toulouse, Hôpital La Grave, Toulouse, France; University of Geneva, Switzerland

## Abstract

**Background:**

Although the reliable and valid Child Post-Traumatic Stress Reaction Index (CPTS-RI) is a widely used measure of posttraumatic stress disorder (PTSD) symptoms in children, it has not been validated in French-speaking populations. The present study aims to assess the psychometric properties of the CPTS-RI in three samples of French-speaking school-children.

**Methods:**

Data was obtained from three samples. Sample 1 was composed of 106 children (mean (SD) age = 11.7(0.7), 50% females) victims of an industrial disaster. Sample 2 was composed of 50 children (mean (SD) age = 10.8(2.6), 44% females) who had received an orthopaedic surgical procedure after an accident. Sample 3 was composed of 106 children (mean (SD) age = 11.7(2.2), 44% females) admitted to an emergency department after a road traffic accident. We tested internal consistency using Cronbach's alpha. We examined test-retest reliability using intraclass correlation coefficient. In order to assess the convergent validity of the French version of the CPTS-RI and the Clinician Administered PTS Scale-Child and Adolescent (CAPS-CA), spearman-correlation coefficient was computed. To verify the validity of the cut-off scores, a ROC curve was constructed which evaluated the sensitivity and specificity of each score compared to the diagnosis with the CAPS-CA. We also used principal components analysis with varimax rotation to study the structure of the French version of the CPTS-RI.

**Results:**

Cronbach's alpha coefficient was 0.87 for the French version of the CPTS-RI. Two-week test-retest intraclass correlation coefficient (n = 30) was 0.67. The French version of the CPTS-RI was well correlated with the CAPS-CA (r = 0.76, p<0.001). Taking the CAPS-CA as the diagnostic reference, with a diagnostic cut-off of >24 for the CPTS-RI, the sensitivity and specificities were 100% and 62.6%, respectively. The French version of the CPTS-RI demonstrated a three-factor structure.

**Conclusions:**

The CPTS-RI is reliable and valid in French-speaking children.

## Introduction

The relevance of a diagnosis of post-traumatic stress disorder (PTSD) in children has been the subject of discussion since the first description of this condition [1].

In 1987 the revised DSM-III [2] took into consideration diagnostic factors specific to children and adolescents. Since then, many studies have confirmed the existence of PTSD in school-age children and adolescents [3], [4] and some have verified the relevance of the DSM-III-R diagnostic criteria [5], [6], [7]. In line with this, the DSM-IV has confirmed that PTSD can occur at any age, including during childhood [8]. However, it appears that children may tend to protect their parents from information concerning the real impact of the trauma [9], [10]. In addition, research has also found that parents often underestimate the post-traumatic reactions of their children [11], [12], [13]. This highlights the importance of directly questioning children and adolescents to evaluate their symptoms of PTSD [14], [15]. In this context, a number of tools have been developed and revised allowing a better assessment of PTSD in children [16].

Although it is sometimes difficult to establish a diagnosis of PTSD, it is important to detect PTSD symptoms early in children and adolescents as untreated symptoms may lead to developmental problems [17], [18]. PTSD can be associated with poorer outcomes including cognitive function, initiative, personality traits, self-esteem and impulse control [19]. Changes in personality have also been described [20], [21] as well as regressive behaviour, a marked tendency to pessimism and the feeling of a foreshortened future [19], [22].

Post-traumatic symptoms can be evaluated using clinical interviews, semi-directive structured interviews, or interviewer rated- or self-rated questionnaires. Of interest, self-rated questionnaires pick out internalised reactions and the consequences of trauma which cannot be identified by observation [23].

Among the self-rated questionnaires, the Child Post-Traumatic Stress Reaction-Index (CPTS-RI) [24] in its English original version, is the best studied and most widely used tools [25], [26], [27] in trauma-exposed children and adolescents. Hawkins et al. [28] reported that the CPTS-RI was the most frequently used tool, 33 times out of 65 in studies evaluating post-traumatic symptoms in five reviews from 1995 to 2004 (*Journal of Clinical Child and Adolescent Psychology, Journal of Consulting and Clinical Psychology, Journal of Paediatric Psychology, Journal of the American Academy of Child and Adolescent Psychiatry*, and *Journal of Traumatic Stress*) [28].

The CPTS-RI is a scale comprised of 20 Likert-type items, intended for children from 6 to 16 years, which evaluates the symptoms of PTSD after exposure to various traumatic events. It was designed to be administered by a clinician, but may also be used as a self-rated questionnaire in children >8-years of age [24], [29], [30]. Each item frequency is rated on a 5-point scale, from never ( = 0) to almost always ( = 4). The global score consists of the sum of the 20 items and ranges from 0 to 80, with higher scores indicating higher PTSD symptom severity. The time required for completion of the scale is 15–20 min.

The scale was one of the first used to measure post-traumatic symptomatology [24], [30]. It is an adaptation of a scale originally developed for adults [31]. While the development was originally based on DSM-III-RW criteria, the DSM diagnostic criteria have undergone some changes since then.

The CPTS-RI is a flexible tool and has been adapted for children or adolescents from different cultures, exposed to various traumatic experiences. It has been translated into many languages (Arabic, Croatian, Kuwaiti, Norwegian, Vietnamese and French). The existence of many studies using different versions of this scale confirms its good adaptation to children of different ages and cultures, or victims of various traumatic events. It has, for example, been used in Armenian children who survived an earthquake [32], in Kuwaiti children who lived through the first Gulf war [33], in Cambodian children who survived the war [34], [35], in American adolescents who were victims of sexual abuse [36], in children who have received bone marrow [37], [38] or liver [39] transplants, and in children victims of road traffic accidents [40], [41].

In general girls tend to give higher scores than boys [42].The CPTS-RI has also been shown to be sensitive to change after treatment including medication (e.g., morphine in children with burns [43] or psychotherapy in a group of adolescents survivors of murder victims [44]).

Even though it is one of the most widely used scales worldwide to evaluate the symptoms of PTSD in children, the psychometric properties of the French version of the CPTS-RI have never been reported nor studied to date. Here, we examine the psychometric properties of the French version of the CPTS-RI using the data from three studies conducted among children who had experienced different traumatic events, and report that this tool is perfectly usable in French-Speaking children and adolescents.

## Methods

### 1. Subjects and procedures

The data used here originated from three studies involving a total of 262 children aged 6–15 years who had been exposed to a DSM-IV A1 qualifying traumatic event. The first study involved children victims of an industrial disaster, the second, children requiring an orthopaedic/trauma intervention, and the third, children victims of a road accident.

The French Consultative Committee for the Protection of Individuals in Biomedical Research of the South West (national ethics committee) approved all procedures of the preset study. For each participant, signed informed consent was obtained from the parents and the child.

#### 1.1. Sample 1

The data used were obtained from a study carried out in 106 children aged 11–14 years [45], [46] in the aftermath of an explosion in a chemical factory on the 21^st^September 2001 in Toulouse, France, which resulted in 30 deaths, 3000 injured, and 30000 damaged or destroyed homes.

One year after the explosion, a cross-sectional study was conducted to evaluate the consequences on mental health in children educated in the 6^th^ grade in five middle schools situated in the disaster area. The children initially completed the Impact of Events Scale (IES) [47]; as a screening procedure. A total of 106 children were contacted for this study: the 50 who scored the lowest scores on the IES, and the 56 who scored the highest. The objective was to compare, children with clinically relevant PTSD symptoms (High IES score) to those with low PTSD symptoms (low IES score), on measures of Child's perception of family cohesion and adaptability, child's experience of the explosion, and parental characteristics [48]. Data collection, including assessment of the CPTS-RI, was carried out 18–24 months after the disaster. The mean age of the children was 11.7 years (±0.7); and 53 of them (50%) were girls.

#### 1.2. Sample 2

The second sample involved a population of 50 children aged 6–15 years who, after an accident, had received an intervention in paediatric orthopaedic surgery at Toulouse University Hospital. PTSD is frequent during the recovery period after paediatric orthopaedic trauma, even among patients with relatively minor injuries [49].

A first CPTS-RI was completed during a consultation following the surgical intervention. It was then proposed to collect information 15 days later with the CPTS-RI completed in the home and returned by post. In order to estimate a test-retest reliability, an interval ranging from 2 days to 2 weeks is generally believed to be a reasonable compromise between recollection bias and unwanted clinical change [50].

The mean age of the participants was 10.8 years (±2.6); and 22 of them (44%) were girls. Among them, 30 (60%) were victims of a fall, 15 (30%) of a sporting accident, four (8%) a bicycle accident and one (2%) of a motor vehicle accident. The mean time between the surgical intervention and completion of the first questionnaire was 29.0 days (±31.4).

Of the 50 children who completed the first questionnaire, 30 participated in the retest. The mean delay between the two tests was 13.5 days (±3.3) (range: 4–20).

#### 1.3. Sample 3

The third sample consisted of children aged 8–15 years who were victims of a road traffic accident and were admitted to the paediatric emergency services of Toulouse University Hospital [46]. The questionnaire was administered 5 weeks after admission to the emergency department.

The mean age of the 106 children who completed the CPTS-RI was 11.7 years (±2.2; range: 7.2–15.6) and 47 of them (44.3%) were girls.

Among these patients, 38 were passengers in a car (35.8%), 32 were pedestrians (30.2%), 21 were on a bicycle (19.8%), 11 were on a motor bike/scooter (10.4%), one on public transport (0.9%) and one in a camper van (0.9%). For 2 of them type of road traffic accident was not specified (1.9%).

### 2. Measures

#### 2.1 French Version of the Child Post-Traumatic Stress Reaction-Index

The French translation was carried out by the group of Philippe Robaey at the Laboratory of Cognitive Psychophysiology and Neuropsychiatry, Sainte-Justine Hospital, Montreal, Quebec (Canada) [51] by the back translation technique. It was adapted to be in line with French language spoken in France by our group (PB and JPR). In particular, we carefully replaced certain French Canadian wordings with sentences more appropriate to a French public, without changing the original meaning. In the three studies, the children completed this French version of the CPTS-RI.

#### 2.2 Clinician Administered PTS Scale-Child and Adolescent (CAPS-CA)

In addition to the CPTS-RI, 103 children from the third sample were assessed with the CAPS-CA [52], [53], 5 weeks after their accident.

The CAPS-CA is a clinician-administered clinical interview comprised of 33 items developed from the adult version of the CAPS. The CAPS-CA takes into account DSM-IV A1 (type of incident) and A2 (immediate reaction of intense fear, helplessness or horror) criteria to evaluate the diagnosis of PTSD. The CAPS-CA evaluates the 17 symptoms of PTSD according to the DSM-IV criteria, as well as eight associated symptoms (guilt, shame, dissociation, changes in attachment behaviour and specific phobias of trauma). Each symptom is rated as a function of its frequency and intensity.

The tool is adapted to children from 8-years to young adolescents. The CAPS-CA enables the diagnosis of PTSD by evaluating the immediate impact of the trauma (A criteria) and the repercussions of the symptoms on development and academic and social functioning. The CAPS-CA is recommended as the ‘gold-standard’ diagnostic tool by international consensus for clinical research protocols [54], however, its assessment is time consuming and required trained clinicians.

### 3. Statistical analyses

Cronbach's alpha was used to assess internal consistency. We examined Test-retest reliability using intraclass correlation. Concurrent validity was determined using Spearman-correlation coefficient, since the data were not normally distributed. Factorial validity was assessed by principal component analysis (PCA) with varimax rotation. The Kaiser-Meyer-Olkin measure [55], an indicator of the appropriateness of the factor solution was computed. To verify the validity of the cut-off scores, a ROC curve was constructed which evaluated the sensitivity and specificity of each score compared to the diagnosis with the CAPS-CA. A total severity cut-off score of 40 points with the CAPS-CA was chosen as it corresponds to the cut-off for clinical diagnosis [53]. Then we analyzed the ROC curve with the CPTS-RI cut-off >24 as recommended by Landolt et al. [40].

The level of statistical significance was set to 0.05 (two-tailed). All statistical analyses were conducted using SPSS 17.0 software [56].

## Results

### 1. Internal consistency

Across the three samples, Cronbach's alpha for the CPTS-RI was high (0.87). Cronbach's alpha for samples 1, 2 and 3, were 0.91, 0.68 and 0.84, respectively.

### 2. Test–retest reliability

Test-retest reliability was determined among the 30 children from sample 2 who completed the second assessment of the CPTS-RI two weeks later.

At baseline, the children who returned the second questionnaire had lower initial CPTS-RI scores (mean CPTS-RI: 14.4±7.4) than those who did not return it (mean CPTS-RI: 20.5±8.9) (Mann Whitney: p = 0.018). The scores at the two assessments were strongly correlated: intraclass correlation coefficient = 0.67 (p<0.001).

### 3. Factor validity

Two-hundred and six children provided data for the 20 items of the CPTS-RI. The case-items ratio was >10, as recommended by Nunnally [57]. The Bartlett test, which tests the null hypothesis according to which all correlations are equal to zero [58], showed that the factor model was appropriate (Bartlett test p<0.001). The Kaiser-Meyer-Olkin measure [55], which is an indicator of the appropriateness of the factor solution, of 0.89, revealed that all of the variables retained are a coherent group and constitute one or several adequate measures of the concept. The minimum value for appropriate factor analysis suggested to be 0.6 [59].

We used principal component analysis (PCA) followed by varimax rotation and in keeping with the scale construct [24] set the model to three factors. PCA of the 20 items explained 44.8% of the variance with three factors (Table 1).

**Table 1:** Principal components analysis (PCA) of three factors and of one factor. Matrix of items.

	PCA of three factors			PCA of one factor	
	Factor 1	Factor 2	Factor 3	Factor 1	
**Item1**	**0.624**	0.068	−0.221	**0.335**	Identified event as Traumatic
**Item2**	**0.639**	0.287	0.272	**0.711**	Negative Emotions
**Item3**	**0.538**	0.493	0.228	**0.751**	Repetitive Images
**Item4**	**0.496**	0.464	0.225	**0.705**	Repetitive Thoughts
**Item5**	0.323	0.392	**0.486**	**0.675**	Dreams and Nightmares
**Item6**	0.453	**0.549**	0.061	**0.652**	Fear of Recurrence
**Item7**	−0.342	0.286	**0.526**	**0.205**	Anhedonia
**Item8**	**0.485**	0.030	0.389	**0.511**	Social Estrangement
**Item9**	**0.525**	0.149	0.397	**0.612**	Emotional Avoidance
**Item10**	0.408	0.145	**0.494**	**0.581**	Emotional Numbing
**Item11**	0.375	**0.520**	0.169	**0.635**	Being Easily Startled
**Item12**	0.178	**0.264**	**0.637**	**0.575**	Sleep Disturbance
**Item13**	0.131	**0.528**	−0.158	**0.331**	Guilt
**Item14**	0.139	**0.520**	0.316	**0.555**	Memory Problems
**Item15**	0.019	0.009	**0.565**	**0.284**	Concentration Difficulties
**Item16**	**0.579**	0.240	0.153	**0.588**	Avoidance
**Item17**	**0.563**	0.486	0.251	**0.773**	Being Upset by Reminders
**Item18**	0.172	−0.002	**0.468**	**0.329**	Behavioral Regression
**Item19**	−0.009	**0.749**	0.087	**0.492**	Somatic Complaints
**Item20**	0.144	**0.473**	0.200	**0.474**	Reckless Behaviors

Items 1, 2, 3, 4, 8, 9, 16 and 17 loaded on the first factor, explained 31.6% of the variance and reflected symptoms of reexperiencing of the event, numbing, disinterest in the outside world and avoidance.

Items 6, 11, 13, 14, 19 and 20, loaded on the second factor, explained 7.3% of the variance, and corresponded to symptoms of fear and anxiety secondary to the event.

Finally, the third factor consisted of items 5, 7, 10, 12, 15 and 18, explained 5.9% of the variance and was related to the difficulties in concentration at school and problems with sleep.

We also conducted a single factor PCA (Table 1), as it may be relevant to only consider a single general factor [60] and use the global score of the tool [51]. In our single factor model with the exception of items 7 and 15, all items exhibited loading factors >0.32 (Table 1).

### 4. Concurrent validity

One hundred and three children from the third sample completed the CPTS-RI and the CAPS-CA 5 weeks after exposure to the trauma. The correlation between the two scales was strong (r = 0.76; p<0.001).

### 5. Receiver operating characteristics (ROC) curve

**Figure 1:**
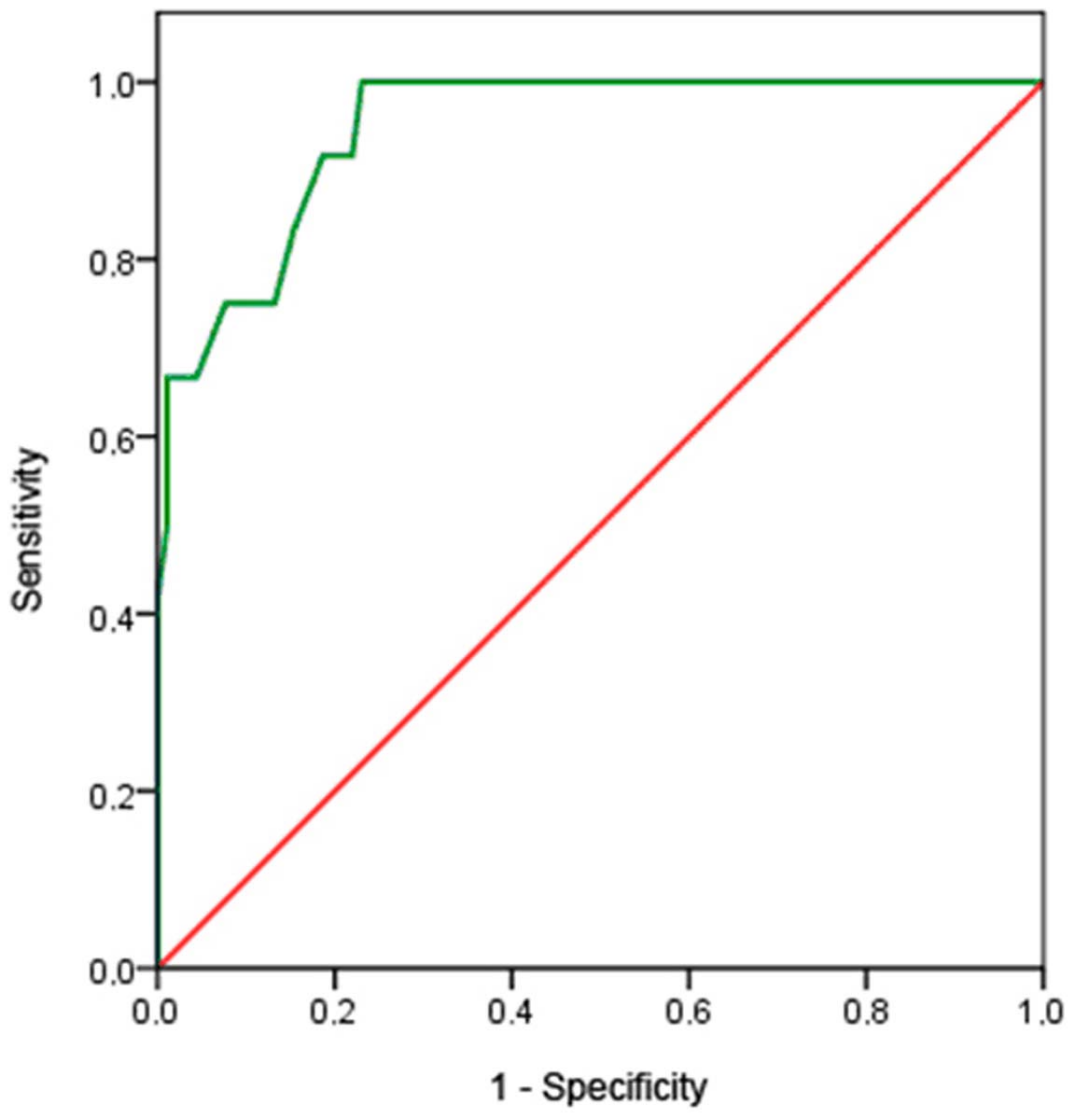
ROC (receiver operating characteristics) curve.

To verify the validity of the cut-off scores, a ROC curve (Figure 1) evaluating the sensitivity and specificity of the scores compared to the diagnosis with the CAPS-CA was constructed. A total severity cut-off score of 40 points with the CAPS-CA was chosen as it corresponds to the cut-off for clinical diagnosis [53].

Taking the CAPS-CA as the diagnostic reference, with a diagnostic cut-off of >24 for the CPTS-RI, as recommended by Landolt [40], the sensitivity and specificities were 100% and 62.6%, respectively. Choosing a higher cut-off score increased the specificity but decreased the sensitivity. The sensitivity and specificity for each cut-off score chosen can be calculated from the ROC curve coordinates (Table 2). The area under the curve was 0.948 (95%CI: 0.897; 0.998 p<0.001).

**Table 2:** ROC (receiver operating characteristics) curve coordinates.

Positive if ≧:	Sensitivity	1-Specificity
0	1	1
1.5	1	0.989
3	1	0.945
5	1	0.923
6.5	1	0.89
7.5	1	0.879
8.5	1	0.857
9.5	1	0.835
10.5	1	0.78
11.5	1	0.758
13	1	0.736
14.5	1	0.714
15.5	1	0.67
16.5	1	0.637
17.5	1	0.604
18.5	1	0.593
19.5	1	0.56
20.5	1	0.505
21.5	1	0.473
22.5	1	0.429
23.5	1	0.407
**24.5**	**1**	**0.374**
**25.5**	**1**	**0.341**
26.5	1	0.275
27.5	1	0.242
28.5	1	0.231
29.5	0.917	0.22
30.5	0.917	0.187
31.5	0.833	0.154
32.5	0.75	0.132
33.5	0.75	0.121
34.5	0.75	0.11
35.5	0.75	0.088
36.5	0.75	0.077
37.5	0.667	0.044
38.5	0.667	0.022
39.5	0.667	0.011
40.5	0.583	0.011
41.5	0.5	0.011
45	0.417	0
48.5	0.167	0
51.5	0.083	0
55	0	0

## Discussion

Our study is the first examination of the psychometric properties of the French version of the CPTS-RI. Overall, our results suggest that the CPTS-RI exhibits satisfactory internal consistence, test-retest reliability, concurrent validity and factorial validity in French-speaking school-aged children, and provide support for its use in this population.

The lowest age of 6-years has been retained since it corresponds to that used to validate the original English version of the scale. Below this age, even if there is a memory of associations, there is not sufficient narrative memory for the child to possess a continuous representation of themselves and their history to respond to a retrospective questionnaire.

The three-factor structure of our French version with factors explaining 31.6%, 7.3% and 5.9% of the variance, respectively, is comparable to that reported for the original version using data for children subjected to a sniper attack in their school [24], [61]. The first factor consists of items exploring re-experiencing of the event, numbing, disinterest in the outside world and avoidance. It explains 31.6% of the variance. Two of the major criteria of the DSM III-R were included in this factor, which was the most important. The second factor included the symptoms of fear and anxiety secondary to the event and represented 7.3% of the variance. The third, which represented 5.9% of the variance, included items related to the difficulties in concentration at school and problems with sleep.

Item 3 (concerning recurrent and intrusive distressing recollections of the event such as images or sounds) and item 4 (unwanted thoughts concerning the traumatic event) are central symptoms of the PTSD and are inevitably correlated, so they loaded on both factor 1 (exploring re-experiencing of the event, numbing, disinterest in the outside world and avoidance), and factor 2 (symptoms of fear and anxiety secondary to the event). Of interest, item 15 (difficulty sustaining attention since the event) did not load highly at the 1-factor solution indicating that that attention deficit may not be as relevant to PTSD in our population of children.

A scale is usually considered to be of interest when the percentage variance explained by the first component is sufficiently large, >30% in the psychosocial domain [62]. In the present case, we observed a variance of 44.8% explained by the three factors, suggesting that the CPTS-RI is a valid tool.

The first step for individuals wanting to use diagnostic tools is to establish appropriate cut-off scores with their related sensitivities and specificities. The first cut-off scores were proposed by the authors of the CPTS-RI: between 7 and 9, PTSD was considered to be mild; between 10 and 12, PTSD was moderate; and >12, PTSD was severe. Using these directives, the correlation between the CPTS-RI and a confirmed clinically diagnosis was 0.91 [31], [63]. Subsequently, the authors of the scale proposed new cut-off values: a total score of 12–24 was associated with a mild level of PTSD, from 25–39 with a moderate level, from 40–59 with a severe level, and ≧60 with a very severe level [32], [64]. With these new directives, Goenjian and Pynoos found a moderate to good sensitivity and specificity for the diagnosis of PTSD after an earthquake in Armenia in 1988. Seventy-eight percent of subjects whose diagnostic criteria corresponded to those of the DSM-III had CPTS-RI scores for severe and very severe categories; 90% of subjects with a score of >40 (severe and very severe) had DSM-III diagnostic criteria for PTSD [32], [64]. Similarly, a significant concordance between CPTS-RI scores and clinical diagnosis was demonstrated in Australian children who were victims of road accidents [41]. For Landolt et al. a cut-off of >24 was clinically significant [40].

For this French version, taking the CAPS-CA as the diagnostic reference, and a diagnostic cut-off with the CPTS-RI of >24 as recommended by Landolt et al. [40] the analysis of the ROC curve showed that the sensitivities and specificities were 100% and 62.6%, respectively. The area under the curve of 0.948 (p<0.001; 95%CI: 0.897–0.998) is particularly satisfying. Specific threshold could be chosen differently with results given in table 2. For example with a cut-off set to 28 we could keep 100% sensitivity but gaining more specificity. Choosing a higher cut-off value increases the specificity as expected but affects the sensitivity.

The diagnosis of PTSD may be difficult to establish during clinical interviews, particularly due to avoidance (lack of sensitivity), whereas when it is recognised it is rarely confused with another problem (good specificity) [1].

In this French version, the internal consistency for the three population studies overall was good (Cronbach's alpha = 0.87) and is comparable to those reported by different authors (between 0.78 and 0.89). Sample 2 presented a lower Cronbach's Alpha (0.68) compared to the other samples.

When we examine in detail available data on the original tool, we find:

- a Cronbach's alpha of 0.78 calculated by Nader and Pynoos during a study in Kuwait after the first Gulf war [33].
- the original version of the CPTS-RI showed a high internal consistency with Cronbach's alpha of 0.81during another study exploring 51 mother-child dyads, 30 months after the first Gulf war [65].
- A Cronbach's alpha of 0.89 given by the evaluation of 568 primary school children 3 months after hurricane Andrew in the USA in 1992 [66].
- A Cronbach's alpha of 0.82 reported by Landolt at 4–6 weeks and 0.79 at 12 months during a study set up to predict the symptoms of PTSD in children who were victims of road traffic accidents [40].
- In the study after a sniper attack, the internal consistency of the different factors was high: Cronbach's alpha was 0.80 for factor 1, 0.69 for factor 2 and 0.68 for factor 3 [24].

Our study reported the good internal consistency of the French version of the CPTS-RI comparable to those measured with the original version.

The test-retest reliability is acceptable with an intraclass correlation coefficient of 0.67 (p<0.001). However, the children who did not return the second questionnaire were those who had the highest scores. One possible explanation is that avoidance symptoms are not frequently reported early on right after the trauma, and progress brought about by treatment might actually improve awareness of these avoiding behaviours/symptoms [1]. To our knowledge, there is no examination of the test-retest reliability of the original version.

After the sniper attack, Nader et al. reported good inter-observer agreement with a concordance of 94% and a Cohen kappa of 0.878 showing good stability [63], but as the CPTS-RI is mainly used as a self-rated questionnaire these considerations are less relevant.

It is important to note that the CPTS-RI items do not all correspond to DSM-IV criteria. Initially, the development of the first version of the CPTS-RI was based on syndromic components corresponding to criteria of the DSM-III-R [2]. This version was an adaptation for children of the « Reaction Index for Adults » [31]. The version that we were interested in was established in 1988. In this version, 17 of the 20 questions corresponded to criteria B, C and D of the DSM, while the other three correspond to ‘associated symptoms’. Questions exploring guilt, for example, do not correspond to any of the three classes of criteria of the DSM, although it is now included in the DSM-5 under alterations in cognitions [67]. Some of CPTS-RI items also refer to subjective feelings whereas DSM criteria address the objective consequences (e.g., the CPTS-RI asks about the loss of pleasure of activities whereas the DSM-III is more interested in the reduction in level of activity). Furthermore, the CPTS-RI does not explore criterion E of the DSM-IV which concerns the duration of symptoms [68]. This illustrates the difficulty, which has not yet been completely resolved, in defining the extent of problems specific to PTSD in adults or those that are related to comorbidities.

Although, the CPTS-RI was not developed to measure the DSM-IV diagnostic criteria for PTSD, but rather those of the DSM-III-R, the diagnostic criteria of PTSD do not differ much between the DSM-III-R and DSM-IV; they only differ slightly in the approach to certain symptoms. It has been shown that the diagnostic validity of the DSM-III, DSM-III-R and DSM-IV criteria is stable even if the effects of modifications to these criteria are difficult to evaluate [5]. Apart from the criteria of reaction to the traumatic event, which the CPTS-RI does not evaluate, the recent DSM-5 attaches more importance to behavioral symptoms which accompany PTSD, and proposes four distinct diagnostic clusters instead of three, across 20 items instead of 17 [67]. Although recent DSM-5 field trials for PTSD [69] provide strong support for the reliability of the new 4-factor model in adults, its reliability in children is still to be assessed. Further, the development of new self-rated measures for based on DSM-5 criteria, and their adaptation to French-Speaking children will take many more years. Finally, as noted above, the CPTS-RI is widely used and need for comparison with previous research warrants its use in the years to come.

Finally, in terms of assessment, there are many sources of information available to investigate psychological problems in children. The circumstances during which a clinician may be led to investigate the symptoms of psychotrauma may be very different according to whether the child does or does not present symptoms early on. The circumstances of these investigations can have considerable consequences on the data collected.

It is important to question the child in the absence of their parents in order that the child does not modulate his/her response in their presence [70]. It is also necessary to question the child's family and particularly their parents [54] who can inform the clinician about symptoms that are present but of which the child is not necessarily aware such as, for example, the child playing repetitive games where ‘reenactment’ evokes some aspects of the traumatic event.

The child may themselves, in their own words and subjectivity, describe the symptoms they are aware of, including feelings of shock, but also their feelings and the consequences on their everyday functioning. The clinician should also decipher the non-verbalized symptoms that the child expresses by their attitude, gestures, behaviour and the themes of their games and drawings. In paediatric psychology, the information verbalized by the patient may sometimes be the most limited, particularly when the child is young.

Besides this general phenomenon, and in the context of PTSD, some of the information identified by the patient may be subjected to a filter of conscious avoidance. The existence of a trauma or recognition of the dramatic nature of an event often leads to a tendency to explain any adaptive difficulties present while economising on an analysis of the different factors involved.

## Conclusions

The investigation of post-traumatic symptoms is often complex in children. In terms of an auto-administered tool, the psychometric properties of the French version of the CPTS-RI, as well as the speed and ease with which it is administered and scored, make this a valuable tool for clinicians and researchers to evaluate the symptoms of PTSD in children and adolescents.

In addition to its diagnostic aspect, the CPTS-RI can be used to quantify the intensity of symptoms. With the DSM, the number of present criteria establishes the diagnosis of PTSD. What matters most to patients however are the distress and impairment, which are often difficult to evaluate with diagnostic tools. Like most similar scales, the CPTS-RI should probably be used to quantify PTSD symptom severity rather than to qualify it. Nonetheless, our results suggest that it can be used reliably among French-speaking school aged children.
